# 14-3-3σ and Its Modulators in Cancer

**DOI:** 10.3390/ph13120441

**Published:** 2020-12-03

**Authors:** Ghazi Aljabal, Beow Keat Yap

**Affiliations:** School of Pharmaceutical Sciences, Universiti Sains Malaysia, Gelugor 11800, Penang, Malaysia; Ghazi.aljabal@student.usm.my

**Keywords:** protein-protein interaction (PPI), 14-3-3σ, dimer, stabilizer, inhibitor, cancer

## Abstract

14-3-3σ is an acidic homodimer protein with more than one hundred different protein partners associated with oncogenic signaling and cell cycle regulation. This review aims to highlight the crucial role of 14-3-3σ in controlling tumor growth and apoptosis and provide a detailed discussion on the structure–activity relationship and binding interactions of the most recent 14-3-3σ protein-protein interaction (PPI) modulators reported to date, which has not been reviewed previously. This includes the new fusicoccanes stabilizers (FC-NAc, DP-005), fragment stabilizers (TCF521-123, TCF521-129, AZ-003, AZ-008), phosphate-based inhibitors (IMP, PLP), peptide inhibitors (2a–d), as well as inhibitors from natural sources (85531185, 95911592). Additionally, this review will also include the discussions of the recent efforts by a different group of researchers for understanding the binding mechanisms of existing 14-3-3σ PPI modulators. The strategies and state-of-the-art techniques applied by various group of researchers in the discovery of a different chemical class of 14-3-3σ modulators for cancer are also briefly discussed in this review, which can be used as a guide in the development of new 14-3-3σ modulators in the near future.

## 1. Introduction

The 14-3-3 proteins are a group of acidic polypeptides that are highly conserved in all eukaryotic cells [1,2,3]. The 14-3-3 family was initially described by Moore & Perez in 1967 as an abundant mammalian brain protein family which took its name based on its elution profile, specifically the fraction number of bovine brain homogenate from diethylaminoethyl (DEAE) cellulose column (14th fraction) and subsequent purified fraction 3.3 from gel electrophoresis [4,5,6,7,8]. The 14-3-3 family comprises seven human isoforms which are named after their respective elution positions on high performance liquid chromatography (HPLC) (β-beta, ε-epsilon, γ-gamma, η-eta, σ-sigma, τ-tau, and ζ-zeta) with at least 500 partners forming protein–protein interaction (PPI) in mammalian cells [9,10,11,12]. Moreover, 14-3-3 proteins have also been detected in non-vertebrate species such as plants and yeasts [13,14,15,16,17]. The overall structure of 14-3-3 proteins is highly conserved among the family members with a molecular mass of approximately 28–30 kDa and isoelectric point of 4–5 [9,18]. Crystal structures of 14-3-3 proteins revealed that they are highly helical with a clamp-like shape dimer. All human 14-3-3 isoforms are expressed as both homo- and heterodimers. The dimer form of 14-3-3 proteins is capable of binding two ligand motifs at the same time, either from the same target or from two different partners [19].

**Figure 1:**
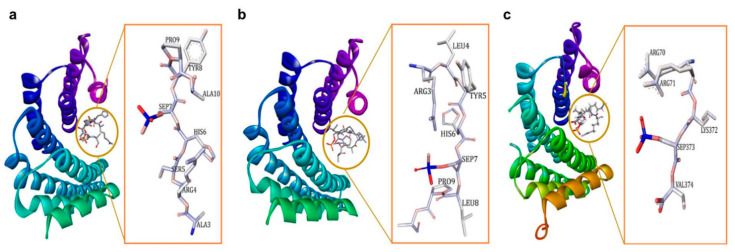
(**a**) 14-3-3ζ/phosphopeptide complex (mode 1, PDB: 1QJB), (**b**) 14-3-3ζ/phosphopeptide complex (mode II, PDB: 1QJA), (**c**) 14-3-3σ/TASK3 peptide (mode III, PDB: 6GHP).

The 14-3-3 proteins are also classified as phosphoserine/phosphothreonine (pSer/pThr)-recognition proteins, as they generally exert their activity through binding to the phosphoserine/phosphothreonine-containing motifs of a multitude of molecules with various functions such as kinases, phosphatases, transmembrane receptors, and transcription factors [2,20,21,22]. In general, there are two high-affinity phosphorylation-dependent binding motifs that are recognized by the amphipathic binding grooves of all 14-3-3 isoforms, i.e., Arg-Ser-Xaa-pSer-Xaa-Pro (R-S-X-pS-X-P, mode I, Figure 1a) and Arg-Xaa-Xaa-Xaa-pSer/Thr-Xaa-Pro (R-X-X-X-pS/T-X-P, mode II, Figure 1b), where X is any amino acid and pS/T represents phosphorylated serine or threonine [23,24,25,26,27]. A third binding motif recognized by the C-terminus of 14-3-3 proteins, i.e., pS/pT-X_1–2_-COOH (mode III, Figure 1c) has also been reported [28,29]. Nevertheless, not all 14-3-3 interactions require a phosphorylated residue as 14-3-3 has also been reported to bind to several non-phosphorylated proteins and peptides, such as exoenzyme S, Cdc25B, and p190RhoGEF [30,31,32,33,34,35].

Consistent with the ability of 14-3-3 proteins to bind to various binding motifs, 14-3-3 proteins are found to be involved in a wide range of physiological processes which include cell proliferation [36,37,38], cell cycle control [39,40,41,42,43], and cell apoptosis [44,45,46,47].

## 2. 14-3-3σ (Stratifin, or Sfn)

The 14-3-3σ protein was first identified in differentiated squamous epithelium by Leffers et al. (1993). 14-3-3σ is unique as it is abundantly expressed in the keratinocytes and epithelial cells [48,49]. 14-3-3σ regulates a wide range of proteins which are mostly involved in oncogenic signaling and cell cycle regulation [50,51]. A comprehensive proteomic study conducted by Benzinger and co-workers found up to 117 proteins associated with 14-3-3σ in human cells, of which the main functional groups include proteins that regulated cytoskeletal organization and dynamics, polarity, adhesion, mitogenic signaling, and motility [52].

**Figure 2:**
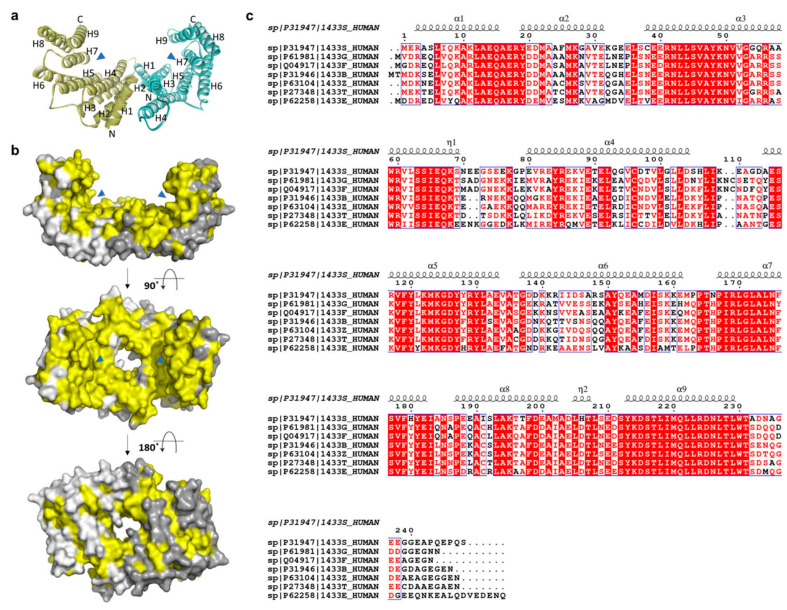
(**a**) Ribbon and (**b**) surface representations of the 14-3-3σ homodimer (PDB: 1YZ5 and 1YWT, respectively). The amphipathic binding grooves are indicated by blue triangles while the conserved residues are highlighted in yellow color. The second and third structures are rotated 90° and 180°, respectively, around the *x*-axis from the previous structure. (**c**) Sequence alignment of the seven human 14-3-3 isoforms (UniProtKB codes, h14-3-3ɛ: P62258; h14-3-3σ: P31947; h14-3-3γ: P61981; h14-3-3η: Q04917; h14-3-3τ: P27348; h14-3-3ζ: P63104; h14-3-3β: P31946), as performed with ClustalW and ESPript 3.0, with red box, white character: strict identity; red character: similarity in group; blue frame: similarity across group. The α-helical regions are indicated above the sequence.

The 14-3-3σ isoform only exists as a homodimer, because the heterodimer form is destabilized by the force of electrostatic interaction between the residue of Glu in position 80 and the residue of Asp or Glu, that replace Ser in position 5 in the 14-3-3σ structure [53,54,55,56]. Like other isoforms, the dimer molecule of 14-3-3σ forms a cup-like shape in which each monomer consists of nine elongated bundles of anti-parallel helices (H1–H9). While four helices (H1–H4) involve in the dimerization with the other monomer, the remaining five helices (H5–H9) form the amphipathic ligand-binding groove (Figure 2a) [53]. Earlier reports indicated that the 14-3-3σ protein can be found in either open (apo form) or closed state when it is bound to its target protein or peptides [57]. While helices H1–H4 were stable in both open and closed state, the intermolecular interactions between the residues in helices H5–H9 were different in the apo-form compared to the bound one. Four hydrophilic residues (Lys49, Arg56, Arg129, and Tyr130) at H3 and H5 have been proposed to contribute to the equilibrium between both states. These residues are believed to form hydrogen bonds with the binding peptide and drive the transit from the open conformation to the closed conformation [57].

The structures of 14-3-3 proteins are highly conserved among all human isoforms. The highest degree of conservation is observed for the amino acid residues lining the amphipathic binding groove. However, major differences were observed in the region adjacent to the amphipathic binding groove and the loop between the residues Ala203 and Asp215 of H8 and H9, respectively (yellow-colored, Figure 2b). These differences illustrate the specific isoform–ligand interaction and subsequently the characteristic biological function of the respective isoforms.

**Figure 3:**
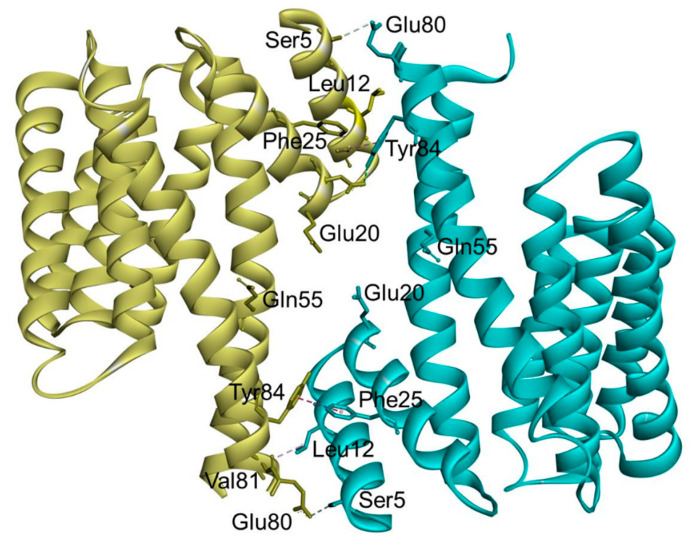
Ribbon representation of the human 14-3-3σ homodimer (PDB: 1YWT). The residues involved in the stabilization of the homodimer molecule and maintaining the full dimerization activity of 14-3-3σ are labeled, with hydrogen bond interactions (cyan dashed line) and hydrophobic interactions (purple dashed line).

In the case of 14-3-3σ, major non-conserved amino acid residues (Ser5, Glu20, Phe25, Gln55, and Glu80) are located at the interface between both monomers near the N-terminus of the 14-3-3σ isoform. Among them, the residues Ser5, Glu20, Phe25, and Glu80 together with the highly conserved hydrophobic core residues located at the dimer interface, i.e., Leu12 and Tyr84, were found to play a crucial rule in the stabilization of the homodimer molecule and maintaining the full dimerization activity of 14-3-3σ (Figure 3) [53,58]. This is evidenced by the dissociation of the dimeric 14-3-3σ into monomers with diminished function upon mutation of these residues [53,58,59].

## 3. Role of 14-3-3σ in Cancer

The 14-3-3σ protein has attracted the attention of researchers as a vital target to fight against cancer growth and metastasis. Previous studies have demonstrated the role of 14-3-3σ in suppressing tumor metabolic reprogramming [60]. In addition, few reports have also highlighted the crucial role of 14-3-3σ against the cancer cell invasion and metastasis. For instance, a low level of 14-3-3σ has been shown to promote production of lactate which stimulates the migration of epithelial cancer cells to a distant organ through breaking down of extracellular matrix [60,61]. Studies have also showed that, among all seven well-known human 14-3-3 isoforms, 14-3-3σ is the only isoform that possesses tumor-suppressing activity [9,19,52,62,63]. It has been shown that 14-3-3σ protein directly controls the G2-M checkpoint of the cell cycle by protecting the tumor suppressor factor P53 against the MDM2-mediated ubiquitination and degradation [64,65,66]. In addition, 14-3-3σ was also reported to play a crucial role in the cell cycle arrest regulation by acting as a cyclin-dependent kinase (Cdk) inhibitor, i.e., through sequestering the cyclin-dependent kinase 1-cyclin B1 complex from entering nucleus and initiate mitosis, as well as binding to the cyclin-dependent kinases 2 and 4 [67,68]. Moreover, 14-3-3σ was also found to negatively regulates the oncogenic activity of the Protein kinase B (also known as Akt) and thus protecting against Akt-mediated tumorigenesis [64]. Further, 14-3-3σ has also been reported as a target gene in mammary epithelial cells which regulates the antiproliferative activity of the transforming growth factor-beta 1 (TGF-b1) through the Smad3-dependent mechanism [69,70]. Furthermore, reports have demonstrated 14-3-3σ involvement in controlling cell proliferation and cancer metastasis via the termination of NF-ĸB signal in mammary cells by regulating the nuclear export of the p65 subunit of NF-ĸB transcription factor and subsequently inhibits its transcriptional activity [71,72]. Moreover, 14-3-3σ has also been reported to regulate the expression of human TASK-3 channel (which is believed to facilitate cancer cell’s proliferation and survival), by blocking the endoplasmic reticulum retention sequences, and thereby promoting the surface expression of this channel [73,74,75]. 14-3-3σ also regulates the oncogenic activity of transcriptional coactivator TAZ which is an oncogenic protein that promotes cell proliferation and migration. The binding of TAZ to 14-3-3σ leads to cytoplasmic retention of TAZ which subsequently disabling its function [76,77].

Unlike other isoforms which show elevated expression in many types of cancer, 14-3-3σ protein level is downregulated in chronic myeloid leukaemia, nasopharyngeal carcinoma, as well as lung, breast, oesophageal, uterine, ovarian, and skin cancers [2,78,79,80,81,82]. The low expression level of 14-3-3σ protein in many cancer types has been linked to either promoter hypermethylation of Sfn gene (which encodes the 14-3-3σ protein) or direct 14-3-3σ degradation through ubiquitination which eventually aborts the normal physiological role of 14-3-3σ against tumor growth and metastasis [62,83,84,85,86]. Consistent with these observations, introduction of a DNA demethylating agent, 5-aza-20-deoxycytidine significantly upregulated the expression level of 14-3-3σ in salivary gland adenoid cystic carcinoma and nasopharyngeal carcinoma [87,88]. In addition, a separate study demonstrated that an upregulation of 14-3-3σ expression by *Marsdenia tenacissima* extract was able to mediate G2/M cell cycle arrest in breast cancer [89].

Although numerous studies have showed the vital role of 14-3-3σ in controlling the tumor formations and metastasis, some studies have also indicated that the 14-3-3σ could be a double-edged sword [79] as its upregulation has also been linked with resistance to chemotherapeutic agents [90,91,92]. In addition, studies have shown that 14-3-3σ also induces overexpression of matrix metalloproteinase 1 (MMP-1), a proteolytic enzyme that degrades native fibrillar collagens, and is often associated with poor prognosis in malignant tumor [79,93,94]. Furthermore, 14-3-3σ has also been reported to bind to the c-Abl protein, preventing its nuclear translocation and subsequently interfering with its pro-apoptotic effect [95,96].

## 4. 14-3-3σ PPI Modulators

As 14-3-3 proteins are capable of binding several hundreds of partner proteins and therefore are involved in the regulation of various cellular functions, a great number of 14-3-3 protein modulators has since been developed in order to address the possibility of modulating the interaction between 14-3-3 proteins and their partner proteins, i.e., either through inhibition or stabilization of their protein-protein interaction [1,97,98]. In this review, we focus to provide an updated overview of the 14-3-3σ modulators that were specifically developed for cancer.

### 4.1. 14-3-3σ PPI Stabilizers

**Figure 4:**
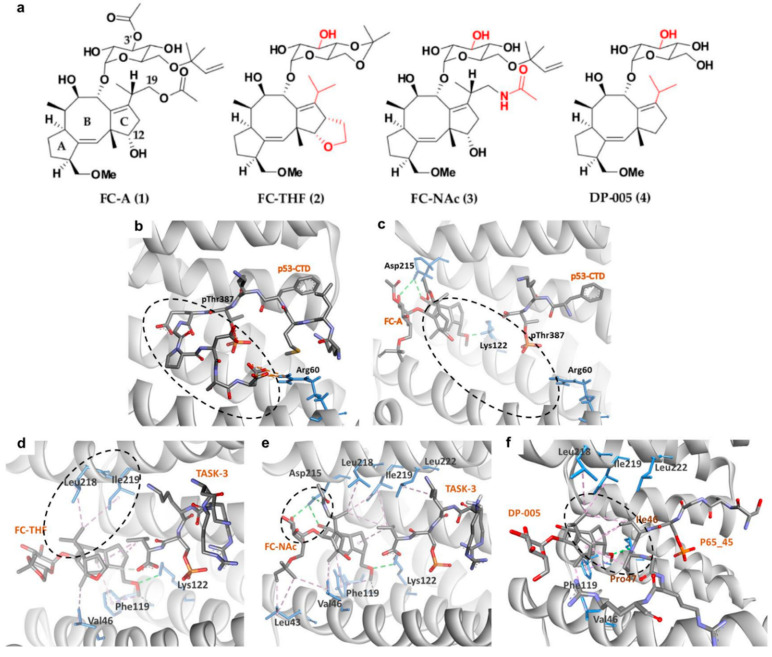
(**a**) Chemical structure of common fusicoccanes stabilizers of 14-3-3σ PPI with the modified groups from compound 1 in red. Three-dimensional (3D) crystal structure of P53/14-3-3σ complex (**b**) in the absence of FC-A (PDB: 5MOC) and (**c**) in the presence of FC-A (PDB: 5MXO), revealed that the C-terminus of the P53 peptide cannot be observed upon binding of FC-A (dashed circles). 3D complex of 14-3-3σ/TASK-3 with (**d**) FC-THF (PDB: 3SMN) and (**e**) FC-NAc (PDB: 6GHP) revealed hydrophobic (purple dashed lines) and hydrogen bond (green dashed lines) interactions between 14-3-3σ and the isopropyl or 19-acetamide moiety of FC-THF and FC-NAc, respectively (dashed circles). (**f**) 14-3-3σ/DP-005/p65_45R ternary complex (PDB: 6NV2) showed that DP-005 not only forms hydrophobic interactions (purple dashed lines) with 14-3-3σ, but also with P65_45 (dashed circle).

With the increasing evidence highlighting the role of 14-3-3σ in suppressing cancer cell growth, metabolism, and metastasis, 14-3-3σ PPI stabilization has begun to gain attention as a promising therapeutic strategy in the discovery of novel bioactive compounds against cancer. In general, PPI stabilizers work as a ‘molecular glue’ to increase the affinity of the partner protein to 14-3-3σ in order to achieve a positive therapeutic effect. An example of a well-studied 14-3-3σ PPI stabilizer is fusicoccin-A (FC-A (1)) (Figure 4a), a diterpene glycoside fungal phytotoxin which was initially found to significantly enhance the interaction between the plant analogue of 14-3-3 and the plant plasma membrane H^+^-ATPase (PMA2) by about 90-fold [99,100]. Recently, compound 1 has been reported to be able to stabilize the interaction between 14-3-3σ and the tumor suppressor gene p53, as confirmed by fluorescence polarization and isothermal titration calorimetry techniques. Nevertheless, a greater disorder in the ternary complex of 14-3-3σ/p53/compound 1 was observed in the crystallographic data where the C-terminus of the peptide is no longer visible (Figure 4b,c), suggesting that either crystal soaking may have forced the p53 peptide to form an unpreferred conformation in the presence of 1 or the fact that 1 acts as an allosteric modulator rather than a ‘molecular glue’ in stabilizing the 14-3-3σ/p53 interaction [101].

Following promising results with FC-A in stabilizing the interactions of 14-3-3σ with its protein partner, semi-synthetic analogue to 1, FC-THF (2) (Figure 4a), which a tetrahydrofuran ring was added to ring C of 1 was generated. However, unlike 1, which stabilizes the interaction of 14-3-3σ with p53, 2 was found to stabilize the interaction between 14-3-3σ and the human potassium channel TASK-3 which is a pro-oncogenic protein that is mainly involved in cancer development. 2 was reported to be able to increase the binding affinity between TASK-3 binding motif and 14-3-3σ protein by up to 19-fold at 100 μM (Figure 4d) (PDB: 3SMN) [74].

To further improve the affinity of fusicoccanes stabilizers on 14-3-3σ, a series of 1 derivatives was designed using molecular dynamic (MD) techniques. As the conserved mode of interaction between 1 and 14-3-3σ is mainly in the form of two hydrogen bonds with Asp215 and hydrophobic interactions with Leu218 and Leu222 of 14-3-3σ, it was hypothesized that presenting a third hydrogen bond with Asp215 would increase the potency of the stabilizer. Therefore, the 19-acetoxy moiety of 1 was replaced with an isostere, 19-acetamide moiety. In addition, the 3′ acetyl group which does not show a significant potency enhancement was also removed from 1 in order to generate a more feasible compound to synthesize. All these modifications led to compound FC-NAc (3), (Figure 4a) which showed enhanced potency and biological activity. Further investigation into the structure of the ternary complex: 14-3-3σ, TASK-3 peptide, and 3 (Figure 4e) (PDB: 6GHP) revealed that Asp215 carboxylate group of 14-3-3σ adopted a new conformation in order to allow the formation of three hydrogen bonds with 3 [102].

Recently, another semisynthetic analogue of 1, DP-005 (4) (Figure 4a) has been reported. Unlike its predecessors which stabilize TASK-3 or p53 interactions with 14-3-3σ, compound 4 acts as a selective 14-3-3/p65 stabilizer. Structural elucidation of the ternary complex p65_45^R^/14-3-3σ/4 (Figure 4f) (PDB: 6NV2) revealed that upon binding of 4, p65_45^R^ peptide adopted a new orientation allowing 4 to form a hydrophobic contact via its isopropyl moiety with Ile46 and Pro47 of the peptide while it binds to Leu218, Ile219, and Leu222 of 14-3-3σ. This additional hydrophobic interaction with p65_45^R^ peptide however is not observed in 1 as the extra 12-hydroxyl group in 1 created a steric and polar clash with the hydrophobic residues of the peptide resulting in unpreferred interactions [72].

**Figure 5:**
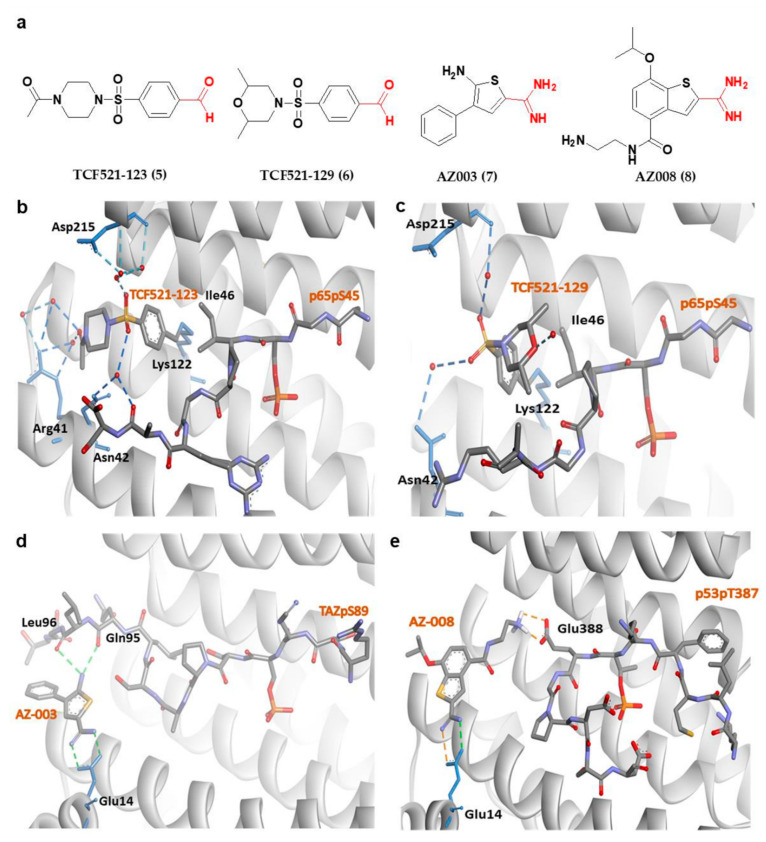
(**a**) Chemical structure of fragment-derived small molecule stabilizers of 14-3-3σ PPI with the key functional groups (i.e., aldehyde and amidine groups) colored in red. (**b**) 14-3-3σ/P65/TCF521-123 ternary complex (PDB: 6YPY). (**c**) 14-3-3σ/P65/TCF521-129 ternary complex (PDB: 6YQ2). (**d**) 14-3-3σ/TAZ/AZ-003 ternary complex (PDB: 6RHC). (**e**) Docked structure of AZ-008/14-3-3σ in complex with p53pT387. Water-mediated hydrogen bonds are shown as blue dashes, while hydrogen bond and ionic interactions are shown as green and orange dashed lines, respectively.

Apart from fusicoccanes stabilizers, other small-molecule stabilizers of 14-3-3σ have also been reported. This include TCF521-123 (5) and TCF521-129 (6) (Figure 5a) which are aldehyde-containing fragment stabilizers of 14-3-3σ/p65 complex. Both 5 and 6 were identified using the site-directed fragment tethering approach whereby the aldehyde-bearing fragments were found to form an imine covalent anchor (instead of hydrogen bond in fusicoccanes stabilizers) to the side chain of Lys122 residue at the amphipathic binding groove. Additionally, the crystallographic data obtained by crystal soaking experiments also revealed a hydrophobic interaction between the aromatic benzaldehyde ring of 5 and 6 with Ile46 of p65, while the sulfonamide group makes additional water-mediated contacts with Asn42 and Asp215 of the 14-3-3σ. However, while the morpholine ring of 6 is engaged directly with p65 peptides, the piperazine moiety of 5 is pointed away from p65 and only engaged in extra water-mediated contacts with both 14-3-3σ and p65 (Figure 5b,c). Nevertheless, both fragments are efficient stabilizers of the 14-3-3σ/p65 complex [103].

Other examples of small-molecule stabilizers of 14-3-3σ are AZ-003 (7) and AZ-008 (8) (Figure 5a), which are fragments with an amidine-containing scaffold. These fragments were identified from fragment-based drug discovery approach in attempts to discover novel compounds to stabilize the interaction between 14-3-3σ and its partners, p53 or transcriptional coactivator, TAZ. However, although 7 was able to interact with 14-3-3σ and TAZ peptide (Figure 5d) (PDB: 6RHC), the stabilization activity could not be confirmed by fluorescence polarization. On the other hand, 8 showed a small overall stabilization of 14-3-3σ/p53pT387 as it preferentially binds to the Glu388 side chain of p53pT387 and forms a salt bridge with the Glu14 carboxylate moiety of 14-3-3σ (Figure 5e) [76].

### 4.2. 14-3-3σ PPI Inhibitors

#### 4.2.1. Phosphonate- and Phosphate-Type Inhibitors of 14-3-3σ

**Figure 6:**
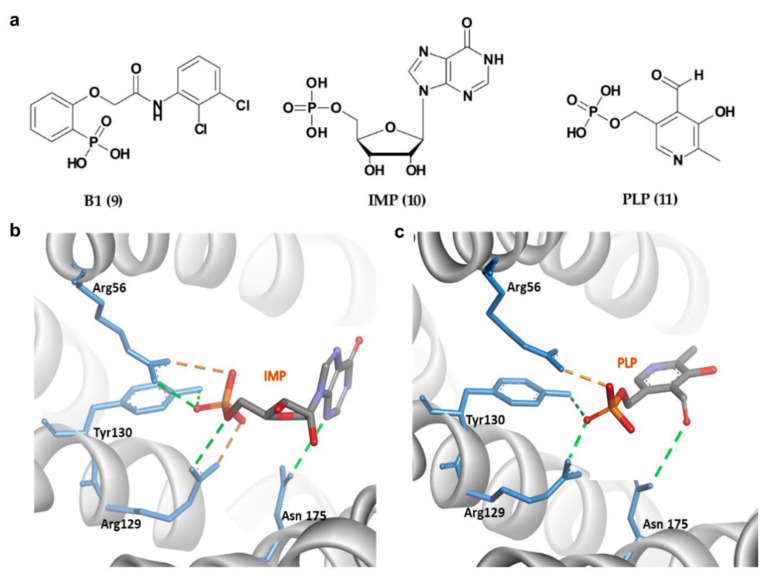
(**a**) Chemical structure of phosphonate- and phosphate-type small molecule inhibitors of 14-3-3σ. (**b**) 14-3-3σ/IMP complex (PDB 6TLF). (**c**) 14-3-3σ/PLP complex (PDB 6TM7). Hydrogen bond and ionic interactions are shown as green and orange dashed lines, respectively.

Apart from 14-3-3σ stabilizers, inhibition of 14-3-3σ interactions with selective protein partners has also received equal attention from the research community in efforts to identify novel therapeutics for cancer. In 2013, Ottmann and his group reported the first exclusively extracellular inhibitors of 14-3-3σ which inhibits the interaction between 14-3-3σ and its membrane receptor aminopeptidase N (APN). APN is required for 14-3-3σ-mediated MMP-1 expression, which its upregulation has been associated with enhanced cancer growth and metastasis [104]. Fourteen potential compounds were identified from the ligand-based virtual screening of about 8 million compounds in the commercial ZINC database and structure-based docking of 512 drug-like initial hits (with phosphonate or phosphate moiety) against the 14-3-3σ amphipathic groove active site. Out of the 14 compounds, compound B1 (9) (Figure 6a) was identified as the most promising inhibitor with the extracellular 14-3-3σ-stimulated MMP-1 levels in human lung fibroblasts downregulated by 9 in a concentration-dependent manner with an IC_50_ of 81 ± 15 μM [94]. Further investigation into the mechanism of interaction between this type of inhibitors and their 14-3-3σ target using molecular dynamic study showed that the phosphate group of the inhibitors existed in an unprotonated state and formed strong hydrogen bonds with the hydrophilic residues (Arg56, Arg129, and Tyr130) inside the amphipathic groove of the 14-3-3σ which largely contributed to their strong binding free energies [105].

In addition to 9, two other phosphate-containing compounds, i.e., inosine monophosphate, IMP (10), and pyridoxal phosphate, PLP (11) (Figure 6a), targeting the amphipathic groove of the 14-3-3σ have also been reported. These compounds were identified via X-ray crystallography and fluorescence polarization assay. Similar to the previously reported phosphate-based inhibitors, the phosphate moiety of 10 and 11 also interacted with the positively charged residues (Arg56, Arg129 and Tyr130) via a H-bonding network (Figure 6b,c). Although both showed a weak inhibitory activity against 14-3-3σ/c-Abl interaction, they were able to localize the cytoplasmic c-Abl into the nucleus in a c-Abl overexpressing cell line at low micromolar concentrations [96].

**Figure 7:**
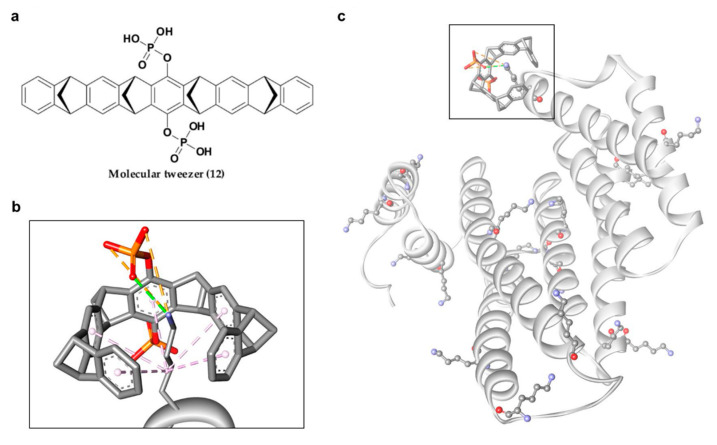
(**a**) Chemical structure of molecular tweezer inhibitor (12). (**b**) Mode of interaction between lysine residue and molecular tweezer inhibitor, with van der Waals interactions (purple dashed lines), hydrogen bond (green dashed line) and ionic interaction (orange dashed lines). (**c**) Representation of the 17 surface lysine residues of 14-3-3σ (ball and stick), with molecular tweezer binding at the periphery (square box).

In 2013, Bier and his group introduced another new phosphonate-type inhibitor of 14-3-3σ, compound 12 (Figure 7a), which is a supramolecular ligand with a belt-like electron-rich molecular cavity formed by alternating fused norbornadiene and benzene rings with a central hydroquinone ring that carries two phosphonate groups [106]. This type of ligand is also classified as molecular tweezer, i.e., a group of artificial receptor molecules with two flat arms that converge to provide a pocket for guest binding [107]. Due to its unique structure, generally only the side chains of lysine and, to a lesser extent, arginine of the target protein would be long enough to be threaded through the tweezer cavity via hydrophobic interactions and form a salt bridge with the anionic phosphate moiety of the tweezer (Figure 7b). As for the 14-3-3σ protein, although 14-3-3σ has 17 surface Lys residues, the tweezer ligand was only found to interact with Lys214 at the edge of the amphipathic binding groove of 14-3-3σ protein and interfere with 14-3-3σ binding to its partner proteins, C-Raf and ExoS. However, such preference for Lys214 by compound 12 over other lysine residues was not observed in a separate study conducted by Shi and co-workers as the molecular dynamics simulation revealed that 10 out of the 17 surface lysine residues were found to have recognition affinity for the molecular tweezer [108]. Nevertheless, both studies observed similar interactions between 12 and 14-3-3σ with the major favorable interactions come from (1) the van der Waals interactions between the long alkyl chain of the lysine residue and the cavity formed by the norbornadiene and benzene rings of 12, and (2) the stable ion pair interactions between the phosphate group of 12 and the positively charged residues on the surface of the protein [108,109]. Intriguingly, unlike other phosphonate-type 14-3-3σ inhibitors which target the central conserved amphipathic groove, compound 12 only binds at the periphery, as evidenced in the co-crystal structures of the molecular tweezer and 14-3-3σ (PDB: 5OEH and 5OEG) [109], suggesting a new interfering mechanism (Figure 7c).

#### 4.2.2. Non-Phosphonate-Type Inhibitors of 14-3-3σ

**Figure 8:**
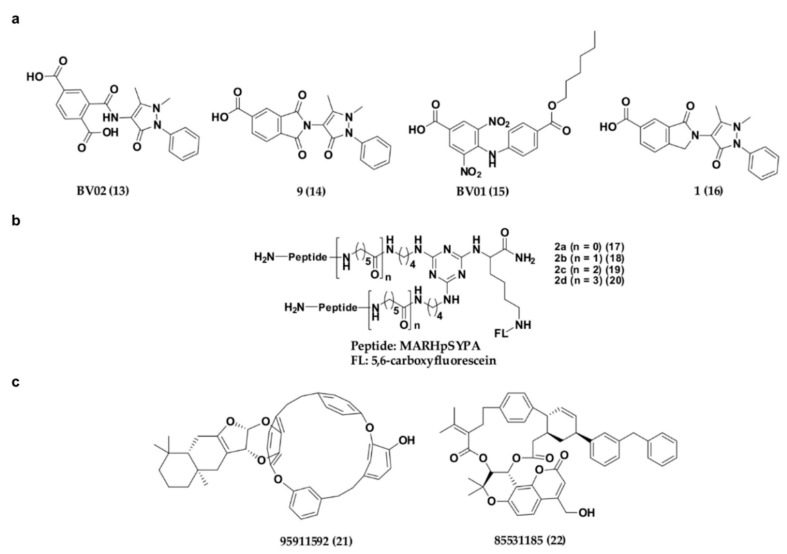
Chemical structure of non-phosphonate-type inhibitors of 14-3-3σ, with (**a**) carboxylate-type small molecule synthetic inhibitors, (**b**) synthetic peptide inhibitors and (**c**) small molecule inhibitors from natural source.

Apart from the phosphonate or phosphate-based inhibitors of 14-3-3σ, a number of research groups have also come up with non-phosphonate-type inhibitors of 14-3-3σ in the past decade. In 2010, Corradi and co-workers has introduced a 14-3-3σ inhibitor, BV02 (13) (Figure 8a) with a remarkable cytotoxic activity (LD_50_ = 1.04 μM) against chronic myeloid leukaemia (CML), which was identified from in silico screening of a commercially available compound library [110]. Compound 13 was reported to be able to disrupt the interaction between 14-3-3σ and c-Abl protein and subsequently promotes c-Abl translocation into the nucleus and provide antiproliferative effects in CML cells expressing the imatinib-resistant T315I Bcr-Abl construct [111,112]. Unfortunately, further studies using NMR techniques showed that 13 undergoes spontaneous chemical rearrangement at room temperature and exists in equilibrium between 2-carbamoyl benzoic form (13) and its bioactive phthalimidic form, 9 (14) [95,113]. To overcome this issue, Corradi and his group used computational techniques, in combination with biophysical and biochemical techniques, to investigate a new set of promising hits with a stable scaffold at room temperature, while Iralde-Lorente and colleagues proposed a synthetic scheme of compound 14 and its chemically stable derivatives. These studies successfully identified two synthesizable and chemically stable compounds, BV01 (15) and 16 (Figure 8a) which showed antiproliferative activity against IM-resistant cells expressing the T315I Bcr-Abl mutation, and a K-562 erythroleukemia cell line at low micromolar concentrations, respectively [95,114].

Recently, a series of bivalent 14-3-3σ peptide inhibitors, 2a–d (17–20) (Figure 8b) were generated using on-resin stepwise substitution reactions on 1,3,5-triazine. While compound 17, which contains the shortest linker, only displays a monomer binding manner (K_D_ = 12.3 μM), compounds with longer linker, 18–20 (K_D_ = 59, 47, 55 nM, respectively) were found to be able to bind to the two identical phosphorylated motifs of 14-3-3σ at the same time and subsequently displayed a 400-fold higher binding affinity and enhanced cellular activity over the monomeric peptide ligand. When compound 19 was conjugated with a cell-penetrating peptide (Arg8) and tested its inhibitory activity against DU145 human prostate cancer cells, the prostate cancer cell growth was effectively suppressed in a dose-dependent manner with minimal cell toxicity [115].

Besides synthetic small molecule and peptide inhibitors, attempt has also been made to identify 14-3-3σ inhibitors from natural source. For example, Shi has recently conducted a virtual screening on the Taiwan natural product database containing more than 20,000 small molecule compounds extracted from 453 Chinese medicine against the crystal structure of 14-3-3σ protein (PDB: 1YZ5). Upon molecular dynamic simulations on the top ranked hits from virtual screening, two compounds, 85531185 (21) and 95911592 (22) (Figure 8c), which contain 16-membered macrocycle and 21-membered macrocycle, respectively, have been proposed to be potential 14-3-3σ inhibitors. Nevertheless, although these compounds were reported to bind in the amphipathic binding groove of 14-3-3σ with strong affinity (with estimated free energy binding values of −10.71 and −15.10 kcal/mol, respectively), their in vitro inhibitory activities however remain to be tested [116].

An overview of all available 14-3-3σ inhibitors for cancer to date with their key targeted amino acid residues on 14-3-3σ were summarized in Table 1. Briefly, all inhibitors (except the molecular tweezers) were found to bind to at least two of the three main residues in the amphipathic groove of 14-3-3σ (Arg56, Arg129 and Tyr130), irrespective of the identity of the protein partners (APN, c-Abl). This suggests that there is a possibility of multi-target inhibition with the current 14-3-3σ inhibitors, resulting in the lack of selectivity of these inhibitors.

## 5. Conclusions

In conclusion, the aberrant expression of 14-3-3σ has been observed in many cancers. Various protein partners and mechanisms involving 14-3-3σ in cancer growth and metastasis have been reported. This suggests that 14-3-3σ is an important target for anticancer drug discovery and development. Consistent with this observation, different chemical classes of 14-3-3σ PPI modulators have been developed as potential therapeutics against cancer. This includes 14-3-3σ PPI stabilizers such as fusicoccanes analogues and fragment-derived small molecule stabilizers, as well as phosphonate and non-phosphonate type 14-3-3σ PPI inhibitors. These modulators were successfully identified using a combination of techniques including in silico tools (ligand-based screening, docking, molecular dynamics simulations), biophysical techniques (NMR, X-ray crystallography, isothermal titration calorimetry), fluorescence polarization, as well as cell-based assays.

However, it is worth noting that both inhibitors and stabilizers of 14-3-3σ PPI available to date mainly target the amphipathic binding pocket. While inhibitors bind directly to the three key amino acids in the amphipathic binding pocket (Arg56, Arg129, and Tyr130), the stabilizers generally bind to the site adjacent to the amphipathic binding pocket, as the amphipathic binding pocket is often occupied by the protein partner of 14-3-3σ. Having said that, a direct interaction with Lys122 at the amphipathic binding pocket of 14-3-3σ was observed in both inhibitors and stabilizers. This suggests that a 14-3-3σ PPI inhibitor is also likely to interfere with the binding of other 14-3-3σ partners which are involved in suppressing cancer cell growth, metabolism, and metastasis, such as the tumor suppressor gene P53, TASK-3, p65, and TAZ. Intriguingly, these amino acid residues are also conserved among all 14-3-3 isoforms. This suggests that modulators that target the amphipathic binding groove of 14-3-3σ may also bind to other isoforms, and may produce other undesirable effects since only 14-3-3σ is frequently downregulated in cancer while other isoforms are usually upregulated.

Although the molecular tweezer seems promising as a potentially selective 14-3-3σ inhibitor as it has been reported to bind to the C-terminal domain of 14-3-3σ, rather than the amphipathic binding pocket, and yet is effective in displacing the binding of the protein partner from 14-3-3σ, it is still unclear if this inhibitor is indeed selective to 14-3-3σ since recent finding seems to suggest that molecular tweezer may binds to any solvently exposed Lys residues. Moreover, the interacting amino acid residue Lys214 is also conserved across all isoforms. Nevertheless, it is clearly demonstrated that it is possible to target other sites on 14-3-3σ in modulating its PPI interaction and is potentially the way forward for the design of new highly selective modulators of 14-3-3σ in the future.

## Figures and Tables

**Table 1:** Comparison between different types of 14-3-3σ inhibitors.

No.	Chemical Classification	Mechanism	IC_50_/% Inhibition/LD_50_/K_D_	Main Residues Involved in the Interaction	References
9	Phosphonate-type inhibitors	14-3-3σ/APN interaction disruption	IC_50_ of 81 ± 15 μM	Arg56, Arg129 and Tyr130, Arg60	[94,104]
10	Phosphate-type inhibitors	14-3-3σ/c-Abl interaction disruption	34% at 1.5 mM	Arg56, Arg129 and Tyr130, Asn175	[96]
11		14-3-3σ/c-Abl interaction disruption	74% at 1.5 mM	Arg56, Arg129 and Tyr130, Asn175	[96]
12		14-3-3σ/C-Raf interaction disruption	480 μM	Lys214	[109]
		14-3-3σ/ExoS interaction disruption	520 μM	Lys214	[109]
13	Carboxylate-type inhibitors	14-3-3σ/c-Abl interaction disruption	LD_50_ = 1.04 μM	Lys49, Arg56, Arg60, Arg129	[95,110,111,112,113,114]
14		14-3-3σ/c-Abl interaction disruption	5.2 ± 0.7 μM	Lys49, Arg56, Arg129, Tyr130, Asn175, Lys122	
15		14-3-3σ/c-Abl interaction disruption	LD_50_ = 1.41 μM	Lys49, Arg56, Arg60, Arg129	
16		14-3-3σ/c-Abl interaction disruption	7.7 ± 2.0 μM	Arg56, Arg129, Lys49, Asn175, Lys122	
17–20	Peptide inhibitors	Disrupting 14-3-3σ interaction with its partners involved in cancer progression	K_D_ 12.3 μM, K_D_ = 59, 47, 55 nM	Targeting the two identical amphipathic grooves	[115]
21–22	Natural products	14-3-3σ/partners interaction disruption	-	Targeting the amphipathic groove	[116]
